# Delivery of Periodontopathogenic Extracellular Vesicles to Brain Monocytes and Microglial IL-6 Promotion by RNA Cargo

**DOI:** 10.3389/fmolb.2020.596366

**Published:** 2020-11-24

**Authors:** Jae Yeong Ha, Song-Yi Choi, Ji Hye Lee, Su-Hyung Hong, Heon-Jin Lee

**Affiliations:** ^1^Department of Microbiology and Immunology, School of Dentistry, Kyungpook National University, Daegu, South Korea; ^2^Department of Oral Pathology, Dental and Life Science Institute, School of Dentistry, Pusan National University, Yangsan, South Korea; ^3^Brain Science and Engineering Institute, Kyungpook National University, Daegu, South Korea

**Keywords:** periodontitis, small RNA, extracellular vesicle, *Aggregatibacter actinomycetemcomitans*, outer membrane vesicle

## Abstract

Gram-negative bacterial extracellular vesicles (EVs), also known as outer membrane vesicles (OMVs), are secreted from bacterial cells and have attracted research attention due to their role in cell-to-cell communication. During OMV secretion, a variety of cargo such as extracellular RNA (exRNA) is loaded into the OMV. The involvement of exRNAs from a range of bacteria has been identified in several diseases, however, their mechanism of action has not been elucidated. We have recently demonstrated that OMVs secreted by the periodontopathogen *Aggregatibacter actinomycetemcomitans* can cross the blood–brain barrier (BBB) and that its exRNA cargo could promote the secretion of proinflammatory cytokines in the brain. However, it was unclear whether the brain immune cells could actually take up bacterial OMVs, which originate outside of the brain, in an appropriate immune response. In the present study, using monocyte-specific live CX3CR1-GFP mice, we visualized OMV-colocalized meningeal macrophages and microglial cells into which bacterial OMVs had been loaded and intravenously injected through tail veins. Our results suggested that meningeal macrophages uptake BBB-crossed OMVs earlier than do cortex microglia. BV2 cells (a murine microglia cell line) and exRNAs were also visualized after OMV treatment and their proinflammatory cytokine levels were observed. Interleukin (IL)-6 and NF-κB of BV2 cells were activated by *A. actinomycetemcomitans* exRNAs but not by OMV DNA cargo. Altogether, these findings indicate that OMVs can successfully deliver exRNAs into brain monocyte/microglial cells and cause neuroinflammation, implicating a novel pathogenic mechanism in neuroinflammatory diseases.

## Introduction

Bacterial extracellular vesicles (EVs), also known as outer membrane vesicles (OMVs) in gram-negative bacteria, are around 10–300 nm sized particles released during all growth stages (Bonnington and Kuehn, 2014). Extracellular RNAs (exRNAs) are RNAs that are incorporated in EVs, which can transport them to other cells. Moreover, as bacterial exRNAs are well protected by EV membranes from the harsh milieu outside of cells, they have been suggested to function as intercellular communication molecules (Choi et al., 2017a; Das et al., 2019). Not only RNAs but also various types of cargo such as proteins, DNA, and lipids are transported by EVs, however, RNAs have gained more research attention due to their sustained biological activities in recipient cells (Lee, 2019). In addition, a recent study suggested the potential involvement of bacterial exRNAs in systemic diseases and their use as biomarkers (Lee, 2020). The majority of exRNAs in OMVs are small RNAs in the size range of 15 and 40 nucleotides (nts) (Ghosal et al., 2015), similar in size to eukaryotic microRNAs (miRNAs) or miRNA-sized small RNAs (msRNAs) in bacteria, implying their regulatory role in host cells (Lee and Hong, 2012; Kang et al., 2013; Blenkiron et al., 2016; Koeppen et al., 2016). Although their precise mechanism of action has not been clearly elucidated, microbial exRNAs are known to have certain roles in host gene regulation, immune response, and diseases (see the review Lee, 2020). For these reasons, the degree of penetration of bacterial EVs to tissues and organs from circulation in the blood from their originated bacterial residency remains an important question to uncover the function of bacterial exRNAs and their relationship with systemic diseases.

A previous short report asserted that intracisternally inoculated *Haemophilus influenzae* type b OMVs could increase the permeability of BBB in rat, which was tested by using radioisotope-labeled albumin (Wispelwey et al., 1989), but it was not clear whether OMVs themselves could actually cross the BBB until our recent demonstration of the evidence (Han et al., 2019). We reported that OMVs from the periodontopathogen *Aggregatibacter actinomycetemcomitans* (*Aa*) could cross the BBB after intracardiac injection, being detectable in the cortex of mice by using brain clearance technique. Furthermore, we observed that exRNAs contained in the *Aa* OMVs activate TNF-α in the mouse brain cortex (Han et al., 2019). However, it was not clear what type of brain cells were responsible for promoting the production of TNF-α by *Aa* exRNAs even though the exRNAs of *Aa* increased the expression of TNF-α in the activated human-macrophage-like cells.

Mononuclear phagocytes, i.e., monocytes and macrophages, are key players in protective immunity and homeostasis (Ginhoux and Jung, 2014). Microglial cells are resident monocytes/macrophages in the central nervous system (CNS) whose primary role as immune defense against infection is critical in modulating neuroinflammation as well as the development of several neurodegenerative disorders such as Alzheimer’s disease (AD) (Block et al., 2007; Ginhoux et al., 2013; Li and Barres, 2018).

In the present study, to explore the neuroinflammatory effect of pathogenic OMVs, we characterize how intravenously injected *Aa* OMVs can be taken up by monocyte-derived cells using intravital imaging analysis of monocyte marker-expressing CX3CR1-GFP knock-in mice. Tail-vein-injected OMVs and exRNA cargo were delivered into meningeal macrophages and cortex microglial cells in a time-sequential manner. Moreover, analysis of the effect of *Aa* exRNAs on the murine microglial BV2 cell line showed that a proinflammatory cytokine other than TNF-α, Interleukin (IL)-6, is promoted in microglia cells by *Aa* exRNAs. We anticipate that our results will shed light on the novel mechanisms underlying immune signals to the brain in response to systemic pathogenic infection.

## Materials and Methods

### Culture

*A. actinomycetemcomitans* (*Aa*, ATCC 33384) was inoculated on brain heart infusion (BHI; Difco, Sparks, MD, United States) agar plates in an anaerobic incubator. After 24 h, the colonies were picked and cultured in BHI media for 48 h. Picked colonies were also checked for ribosomal DNA to avoid any contamination. The anaerobic incubator was maintained at 37°C in 5% CO_2_, 5% H_2_, and 90% N_2_. *Aa* were grown anaerobically in BHI until the desired optical density (OD) was reached (around 0.7 of OD_600_).

Supernatants were collected for OMV purification and bacterial pellets were collected for RNA isolation and purity evaluation.

The BV2 cells, mouse macrophage-like microglial brain cells, were maintained on 6-well plates with 5× 10^5^ cells per well in DMEM medium supplemented with 10% FBS and 100 IU/ml penicillin G. The complete medium was changed three times a week.

### Animals

The G-protein-coupled receptor CX3CR1 is expressed in human monocytes, and CX3CR1-GFP transgenic mice are widely used in studies on microglial cells (Jung et al., 2000). The CX3CR1-GFP mice (B6.Cg-Ptprca Cx3cr1tm1Litt/LittJ, The Jackson Laboratory, Bar Harbor, ME, United States) were housed in accordance with the standard guidelines for the care and use of laboratory animals, the animal protocols having been approved by the Institutional Animal Care and Use Committee (IACUC) of the Korean Advanced Institute of Science and Technology (KAIST, Daejeon, South Korea) for the company IVIM Technology (Daejeon, South Korea). All mice underwent surgery under anesthesia, and all efforts were made to minimize their suffering. Mice were individually housed in ventilated, temperature- and humidity-controlled cages (22.5°C, 52.5%) under a 12:12 h light:dark cycle and provided with standard diet and water ad libitum.

### OMV Sample Preparation

*Aa* OMV samples were prepared as previously described (Han et al., 2019). Briefly, OMVs were obtained from the filtered and concentrated supernatant using ExoBacteria OMV Isolation Kits (SBI, Mountain View, CA, United States) according to the manufacturer’s protocol. The isolated OMVs were treated with 1 μl of RNase A (1 U/μl, Thermo Fisher Scientific, Wilmington, DE, United States) and DNase I (2 U/μl, Thermo Fisher Scientific) to make a 1.5 ml volume of the OMVs, which was then incubated at 37°C for 25 min. This was done to remove any residual nucleic acids in the OMV preparation. The OMVs were purified again using the ExoBacteria OMV Isolation Kits to get rid of unnecessary remained enzymes. Transmission electron micrographs (TEM) of *Aa* OMVs (pre-purification and purified) were compared in Supplementary Figure S1.

For physical lysis, the OMVs were frozen and thawed five times followed by sonication using a sonicator (100 W and 20 KHz; KSC-100 portable ultrasonicator, Korea Process Technology, Seoul, South Korea) for 30 s and then placed on ice for 30 s. Subsequently, 1 μl of RNase A and/or DNase I was added to 1 ml of the lysed OMVs and incubated at 37°C for 25 min. The purified OMVs were checked for sterility by spreading 10 μl of the purified OMVs on BHI agar plates for 3–4 days and stored at −80°C until use.

For staining, the OMVs (resuspended in 100 μl in PBS, without calcium and magnesium; GE Healthcare Bio-Sciences, Pittsburgh, PA, United States) were incubated with 2 μM SYTO RNASelect Green Fluorescent Cell Stain Kit (Thermo Fisher Scientific) and/or 10 μM red fluorescent Lipophilic Tracer DiD (1,1’-dioctadecyl-3, 3, 39, 39-tetramethylindodicarbocyanine, 4-chlorobenzenesulfonate salt; Thermo Fisher Scientific) for 1 h at 37°C. The samples were washed once with PBS followed by ultracentrifugation at 150,000 × *g* for 1 h at 4°C.

Both intact OMVs and lysed OMVs were analyzed for lipopolysaccharide (LPS) concentrations, for which the Pierce LAL Chromogenic Endotoxin Quantitation Kit (Thermo Fisher Scientific) was used according to the manufacturer’s instructions.

### OMV Analysis

The OMVs underwent nanoparticle tracking analysis using the NanoSight system (NanoSight NS300; Malvern Panalytical, Malvern, United Kingdom) according to the manufacturer’s protocols. Samples were diluted 100 times in a total volume of 1 ml of PBS. PBS was introduced into the chamber using a 1 ml syringe and assessed whether it was free from particles and whether the chamber was clean (i.e., no light scattering). Particle size was measured based on Brownian movement and particle concentration was quantified in each sample. Protein quantification of OMVs and OMV lysates were done using Bradford reagent (Bio-Rad, Hercules, CA, United States) and the NanoSight system as previously described (Han et al., 2019).

### Intravital Imaging

**FIGURE 1:**
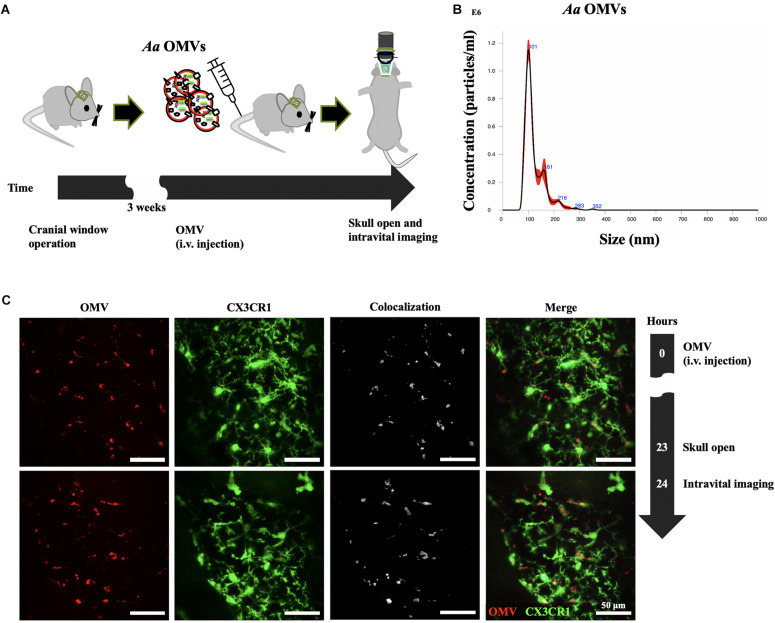
Delivery of *Aa* OMVs into microglial cells. **(A)** Schematic diagram of intravital imaging process performed in this study. **(B)** Nanoparticle tracking analysis (NTA) of *Aa* OMVs used in this study. OMVs from *Aa* were analyzed using the NanoSight system to visualize the size distribution (*X*-axis) and particle numbers (*Y*-axis). **(C)** Intravital image was captured 24 h after *Aa* OMV i.v. injection. OMVs were colocalized with GFP-positive microglial cells. Scale bar: 50 μm. Additional Figures can be found in Supplementary Figure S3.

Three weeks before intravital imaging, cranial window surgery of CX3CR1-GFP mice was accomplished according to a previously published protocol (Holtmaat et al., 2009). Briefly, the skin and skull were removed followed by drilling to remove the cranial bone. The exposed brain was covered by glass, and dental cement was applied on the top of the skull and part of the cover glass, sealing off the exterior. All the exposed skull and wound edges were covered with dental cement. The strategy used for the intravital image acquisition of *Aa* OMV-injected mice is depicted in Figure 1A. Mice were housed for 3 weeks for stabilization before conducting intravital imaging.

After performing mouse tail vein (intravenous, i.v.) injections of DiD-stained OMVs (100 μl; approximately 3.0 × 10^8^ particles) and anti-CD31 monoclonal antibody (60 μl; 553708, BD Bioscience) conjugated with Alexa Fluor 555 (A- 20009, Invitrogen), a laser scanning intravital confocal microscope (IVM-C, IVIM Technology) with an objective lens (Nikon, Japan; magnifications: × 25; numerical aperture: 1.1) was used to visualize the brain cellular uptake of OMVs. During the intravital imaging, the body temperature of the mouse was maintained at 37°C using a homeothermic controller. Mice were anesthetized using intramuscular injections of a cocktail mixture of zoletil (30 mg/kg) and xylazine (10 mg/kg). The imaging analysis was conducted as previously described (Ahn et al., 2017). All experiments were performed in duplicate using two independent OMV sample preparations and at least two mice for each experiments.

All the intravital imaging experiments conducted using mice were performed by IVIM Technology according to institutional and national guidelines.

### Confocal Microscopic Imaging

The OMVs and RNAs inside were stained as described earlier in the OMV sample preparation section of “Materials and Methods.” Stained OMVs were added to BV2 cells cultured on chamber slides and incubated for 24 h at 37°C, then washed four times with 500 μl of PBS, followed by incubation with DAPI (Vector Laboratories, Burlingame, CA, United States). The stained OMVs with the added cells were visualized on a laser scanning confocal microscope (LSM Zeiss 800; Carl Zeiss Microscopy, Jena, Germany) equipped with an objective lens (magnifications: × 40 with water immersion; numerical aperture: 1.2; resolution: 0. 28 μm).

### Total RNA Extraction

Total RNA was extracted using both TRIzol (Invitrogen, Carlsbad, CA, United States) and the miRNeasy Mini Kit (QIAGEN, Valencia, CA, United States) according to the manufacturers’ protocols, with some modifications. Each sample was resuspended in 300 μl TRIzol reagent (Invitrogen). Resuspension was homogenized for 30 s using a Kontes Pellet Pestle Cordless Motor (Sigma-Aldrich, St. Louis, MO, United States) and placed on ice for 30 s, the steps being repeated 5 times for full homogenization. Then, an additional 700 μl TRIzol was added to the samples and incubated at room temperature for 5 min. 1-bromo-3-chloropropane (200 μl, Sigma-Aldrich) was then added to each sample, followed by incubation for 3 min. Next, the samples were centrifuged at 13,499 × g for 15 min at 4°C using a centrifuge (Smart R17 plus, Hanil Science, Incheon, South Korea). The supernatant (approximately 750 μl) of each sample was mixed with 750 μl of 100% isopropanol. The mixed samples were incubated at -20°C for 2 h. The columns of miRNeasy Mini Kit (QIAGEN) were additionally used to purify the small RNA-enriched RNA and clean RNA according to the manufacturer’s protocol.

### qRT-PCR

Total RNA (1 μg) was reverse-transcribed using OmniScript (QIAGEN). The qRT-PCR was performed with diluted cDNA using primer sets for TNF-α, IL-1β, IL-6, and β-actin (Supplementary Table S1). The PCR was performed in 96-well plates using the 7,500 Real-Time PCR System (Applied Biosystems, Foster City, CA, United States). The expression of each gene was determined from three replicates in a single qRT-PCR experiment.

### Immunoassays of Cytokines

BV2 cells were incubated with purified OMVs for 24 h, after which the filtered supernatants using 0.22 μm pore filter (Sigma-Aldrich) were analyzed for four cytokines (IL-1β, IL-6, TNF-α, and IFN-γ) using a quantitative multiplex ELISA (Mouse Cytokine Panel 2 (4-plex); QUANSYS Biosciences, Logan, UT, United States) according to the manufacturer’s instruction. Data were analyzed using the Q-View Software (Quansys Biosciences). All experiments were conducted in triplicate.

### Western Blotting

For the western blotting of NF-κB p65 and phospho-p65, 20 μg of whole cell lysates was used for SDS polyacrylamide gel-running, followed by transfer to a polyvinylidene difluoride (PVDF) membrane using Pierce Power Blotter (Thermo Fisher Scientific) at 110 V (80 mA) for 80 min. The membrane was incubated in 5% skim milk/0.1% TBS-Tween 20 at room temperature for 1 h, followed by incubation with a 1:1,000 final diluted NF-κB p65 (#8242, Cell Signaling Technology) and phospho-p65 antibody (Ser536, #3033, Cell Signaling Technology) and then an anti-rabbit secondary antibody conjugated with horseradish peroxidase (#7074, Cell Signaling Technology). The control for loading was assessed using a β-actin antibody (at a dilution of 1:1,000, SC-47778, Santa Cruz Biotechnology Inc., Santa Cruz, CA, United States).

### Statistical Analysis

All data are presented as mean ± SD. Differences among sample group values were analyzed using a one-way ANOVA and *post hoc* analyses were performed using the Tukey Test. All analyses were conducted using Origin 8.0 software (OriginLab, Northampton, MA, United States), and *p*< 0.05 were considered to be statistically significant.

## Results

### Intravital Imaging Reveals That Tail-Vein-Injected *Aa* OMVs Can Enter Mouse Brain Monocytes

Unlike eukaryotic EV (exosomes), which have been shown to deliver cargo to the brain after crossing BBB (Matsumoto et al., 2017; Yuan et al., 2017), little is known about the ability of peripheral bacterial EVs to reach the brain; we recently suggested that *Aa* exRNAs are the primary causative agents promoting the secretion of proinflammatory cytokines in macrophages and the mouse brain (Han et al., 2019). We therefore intended to confirm, using intravital imaging technique, whether brain monocytes or microglial cells can take up bacterial OMVs and their cargo when injected through the tail vein.

To investigate whether *Aa* OMVs can be taken up by mouse microglial cells, we first investigated the *Aa* OMVs injected (tail i.v. injection) into monocyte-specific CX3CR1-GFP mice using the intravital imaging system (Figure 1A). The number and size distribution of *Aa* OMVs used in this study were analyzed using a nanoparticle tracking analysis system. The majority of OMVs were in the size range of 100–280 nm (Figure 1B). As the NTA system could not precisely detect particle sizes < 60 nm (Bachurski et al., 2019), some smaller OMVs of *Aa* might have been missed. Therefore, the actual particle numbers might be greater than the measurement results we present here. Our previous study found that *Aa* OMVs apparently crossed the mouse BBB after 24 h of intracardiac injection (Han et al., 2019). To reduce the risk of cardiac damage, we switched to i.v. tail injections to administer stained *Aa* OMVs. Membrane-stained (red) OMVs were colocalized with GFP+ microglial cells 24 h after the injection of *Aa* OMVs into the cortex (Figure 1C and Supplementary Figure S3).

**FIGURE 2:**
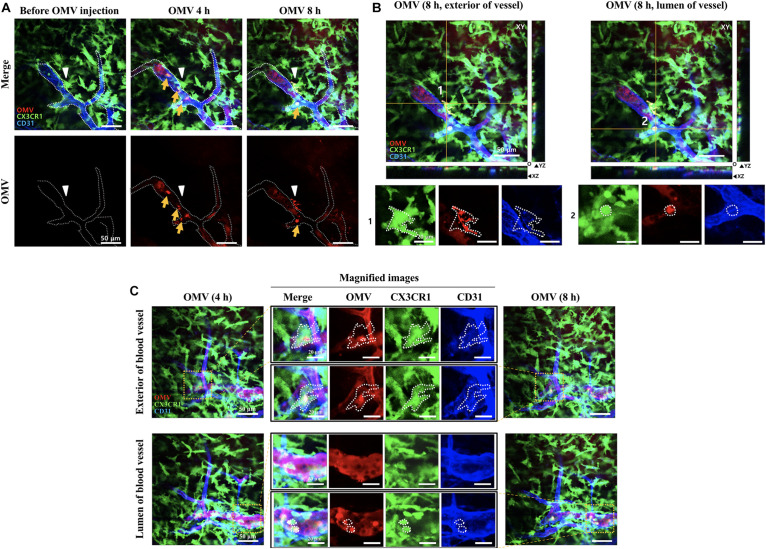
Visualization of tail-vein-injected OMVs and meningeal macrophages of live CX3CR1-GFP mice. **(A,B)** OMVs taken up by monocytes were compared between 4 and 8 h after OMV injection. Confocal microscopic acquisition results show the OMV distribution before and 4 and 8 h after OMV i.v. injection of mice. **(A)** The white dotted line delineates the lumen of blood vessels. White arrowheads indicate *Aa* OMV-colocalized meningeal macrophages, and yellow arrows indicate OMV-colocalized monocytes/macrophages in the lumen of vessel. Scale bar: 50 μm. **(B)** Representative image stacks (5 μm intervals) acquired during confocal imaging of mice with GFP-positive microglial cells for OMVs injected (8 h) shown in **(A)**. The white dotted area refers to the colocalization of OMVs (red) and CX3CR1-GFP-positive monocytes (green). Scale bar: 50 μm; magnified scale: 20 μm. **(C)** Colocalized monocytes/macrophages (dotted areas in the magnified images) in the exterior and lumen of the meningeal blood vessel were compared. Magnified images (scale bar: 20 μm) of colocalized monocytes (shown in yellow in the merged images) in the dotted area are clearly seen in both the exterior and lumen of the meningeal blood vessel.

To ensure detection of localized of OMVs, brain blood vessels were also fluorescently labeled by i.v. injection of anti-CD31 antibody 1 h before imaging. At an imaging depth of around 80 μm (from surface of the brain), where mouse meninges are located, the GFP-positive meningeal monocytes/macrophages were first identified using intravital imaging analysis. As shown in Figure 2A, the majority of OMVs in the meningeal blood vessel lumen are observed at 4 h, suggesting that tail-injected OMVs can disseminate into several organs through blood vessels. OMVs were taken up by GFP-positive “patrolling” monocytes/macrophages in the lumen of vessel (yellow arrow), whereas some OMVs began to cross the blood vessel wall and colocalize with meningeal macrophages at 8 h (white arrowhead in Figure 2A). The magnified and stack images of monocytes/macrophages shown in Figure 2A at 8 h clearly demonstrate the OMVs and GFP-positive cell colocalization (Figure 2B). Another region of colocalized monocytes/macrophages (the dotted area in the magnified images) inside and outside of the meningeal blood vessel was also observed at 4–8 h, as seen in the magnified images in the region marked with yellow squares (Figure 2C). Colocalized monocytes (marked in yellow in the merged images) in the dotted area are clearly observed in both the exterior and lumen of the meningeal blood vessel.

**FIGURE 3:**
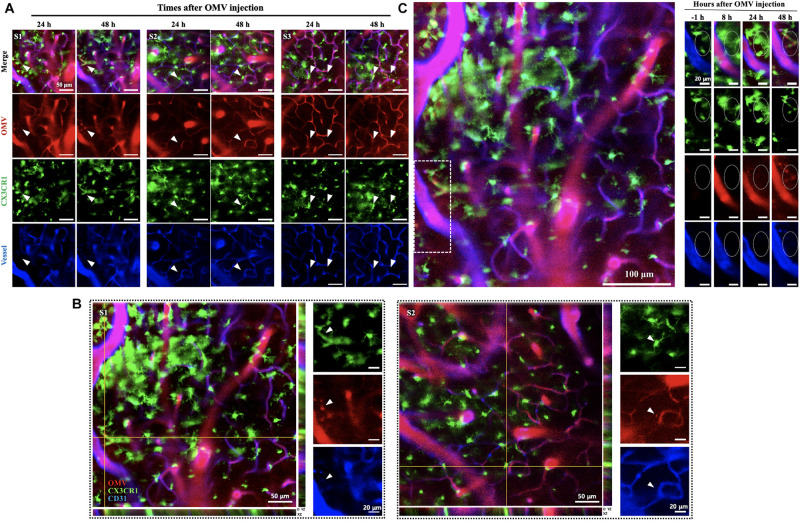
Visualization of tail-vein-injected OMVs and microglial cells in the cortex of live CX3CR1-GFP mice. **(A)** OMVs taken up by microglial cells were imaged at 24 and 48 h after OMV injection. White arrowheads indicate OMVs taken up by microglial cells. Fluorescence-dye-conjugated anti-CD31 antibody was injected 1 h before imaging. Three different regions were captured. Scale bar: 50 μm. **(B)** Representative image stacks (5 μm intervals) acquired during confocal imaging of experimental mice with GFP-positive microglial cells for OMVs injected (48 h). The arrowheads refer to the colocalization of BBB-crossed OMVs (red) and CX3CR1-positive microglial cells (green) localized in the exterior of vessels. Scale bar: 50 μm; magnified scale: 20 μm. **(C)** Confocal microscopic acquisition results showing the OMV distribution before OMV injection (–1 h) and 8, 24, and 48 h after OMV i.v. injection of mice. The white dotted rectangle in the left panel was magnified. Right panel: magnified images of OMVs taken up by microglial cells (dotted circles). Scale bar: 100 μm; magnified scale: 20 μm.

To observe whether the injected OMVs can reach microglial cells, intravital images of cortex were captured at 24 and 48 h as more time was required for observation. Three random field images (Figure 3A) revealed that the OMVs (red) taken up by GFP-positive microglial cells (Figure 3A, arrowheads). Moreover, in the magnified stack images acquired during confocal imaging shown in Figure 3A, the OMV-colocalized microglial cells are clearly visualized in the exterior region of the vessel at 48 h (Figure 3B, arrowheads of S1 and S2 random fields). Although we could not observe whether the OMVs had crossed the BBB and colocalized with cortex microglial cells at 8 h of OMV injection, it was assumed that OMVs that were i.v. injected into the tail crossed the BBB and reached the cortex sometime after 8 h (Figure 3C). These data demonstrate that OMVs can pass through the BBB and internalize into meningeal macrophages, thus allowing cargo into microglial cells of the brain.

### *Aa* OMVs Are Delivered Into the BV2 Microglial Cells

**FIGURE 4:**
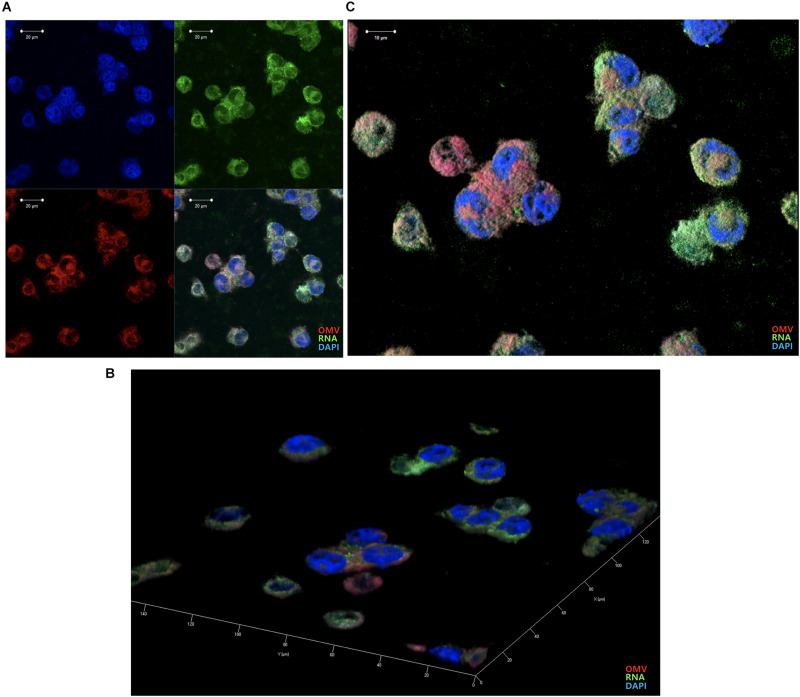
Delivery of *Aa* OMVs and RNA into BV2 cells. *Aa* OMVs were prestained with the lipid tracer dye DiD (red) and RNA-specific dye SYTO RNASelect (green). Stained OMVs (approximately 4.5 × 10^8^ particles) were incubated with BV2 cells on a chamber slide for 24 h at 37°C. DAPI was also counterstained with DAPI (blue) to visualize the nuclei. **(A)** Confocal microscopy analysis of *Aa* OMVs revealed colocalized OMVs and the RNA cargo inside (overlay). Bar = 20 μm. **(B,C)** 3D rendering of confocal fluorescence images. Bar = 10 μm.

We previously reported that *Aa* OMVs can penetrate macrophage cells along with their RNA cargo (Han et al., 2019). Due to differences between macrophages and microglial cells, we repeated the confocal microscopy analysis of OMVs with the RNA cargo. As the SYTO RNA-Select reagent is membrane-permeable, we could detect RNA colocalization with the OMV membrane inside BV2 cells (Figure 4). The stained OMVs along with the RNA cargo were incubated with BV2 cells for 24 h, followed by counterstaining with DAPI to visualize the nuclei. Observation indicated OMVs and RNA cargo can internalize microglial cells, consistent with previous studies conducted using other cell types (Ho et al., 2015; Blenkiron et al., 2016; Koeppen et al., 2016; Choi et al., 2017a; Han et al., 2019).

### *Aa* exRNAs in OMVs Activate IL-6 in BV2 Microglial Cells Through NF-κB Activation

To explore the effect of *Aa* OMVs and the RNAs in OMVs on BV2 cells, we treated BV2 cells with *Aa* OMVs and OMV lysate for 16 h (the LPS levels being approximately 50 ng/ml in both OMV and OMV lysates). We infected around 4.5 × 10^8^ particles of *Aa* OMVs to the cells (5 × 10^5^ cells), the higher number of OMVs resulting in cell death under our experimental condition (cell viability results are shown in Supplementary Figure S2).

**FIGURE 5:**
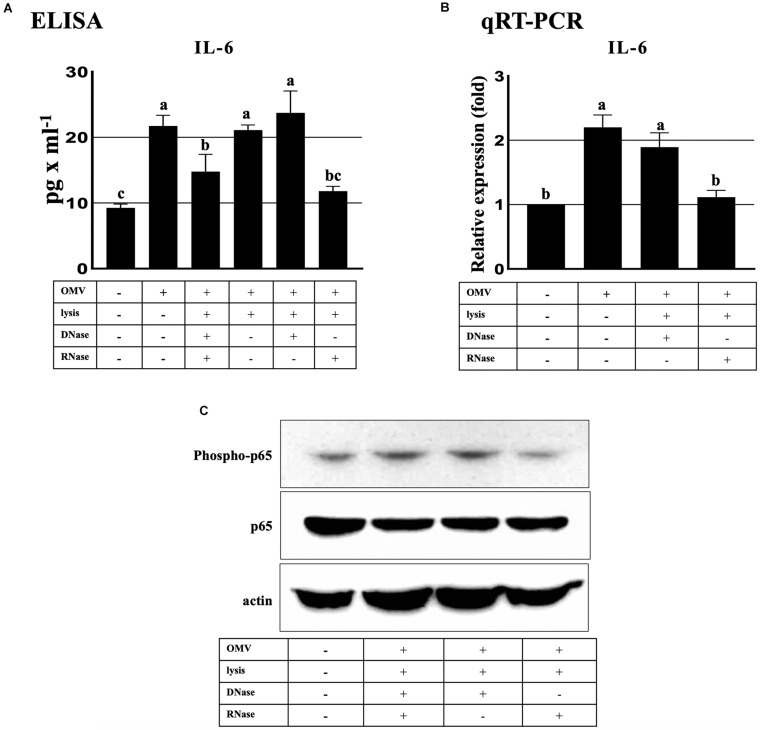
ExRNAs of *Aa* activate IL-6 in BV2 cells through the NF-κB signaling pathway. **(A)** Secreted IL-6 protein levels were upregulated at 16 h after treatment with intact OMVs. IL-6 secretion by OMV lysate was decreased by RNase-only treated OMV lysates in BV2 cells at 16 h. **(B)** qRT-PCR analysis revealed that the transcript levels of IL-6 activation by *Aa* OMV lysates were decreased by RNase-only pretreatment at 16 h. **(C)** NF-κB activation (phosopho-p65, upper panel) was significantly decreased by RNase-only-treated OMV lysates compared with DNase-treated OMV lysates at 16 h. Total NF-κB p65 (middle panel) and actin (bottom panel) levels were assessed for controls. BV2 cells were seeded onto 6-well plates (5 × 10^5^ cells/well) and treated with *Aa* OMVs (approximately 4.5 × 10^8^ particles/well in 2 ml of media) and OMV lysates (the same amount of proteins as in 4.5 × 10^8^ OMV particles). Data are presented as mean ± SD from three independent experiments. The letters (a–c) indicate significant differences at *p* < 0.05.

We performed 4-flex cytokine (IL-1β, IL-6, TNF-α, and IFN-γ) arrays from the BV2 cells treated with *Aa* OMVs and OMV lysate to investigate the effect of *Aa* OMVs and exRNAs on microglial cells. The levels of IL-1β and IFN-γ were too low to detect, whereas IL-6 and TNF-α levels were detectable. However, unlike macrophage-like U937 cells (Han et al., 2019), TNF-α was not detectable in the BV2 cells treated OMVs and OMV lysates (Supplementary Table S2). The level of IL-6 protein measured from accumulated culture media of BV2 cells was upregulated by intact OMVs but not in OMV lysates treated with nucleases (DNase and RNase). This result indicated that the nucleic acid cargo of *Aa* OMVs is responsible for the activation of IL-6. Moreover, OMV lysates were treated with DNase or RNase to examine whether DNA or RNA is further responsible for the elevated IL-6 secretion. The result indicated that RNA rather than DNA cargo is the pivotal factor for IL-6 promotion because the OMV lysate treated with only RNase demonstrated less IL-6 secretion than that treated with only DNase (Figure 5A). Similar results were obtained at the RNA transcript level of IL-6, which was measured by qRT-PCR (Figure 5B).

The human IL-6 gene promoter possesses a binding site for NF-κB, which activates the expression of IL-6 [known to be induced by various stimuli such as LPS, TNF-α, and Poly (I:C) (Tanaka et al., 2014; Kumar, 2019)]. Our previous investigation demonstrated that OMV RNA of *Aa* promoted the activation of NF-κB via TLR8 in macrophage U937 cells (Han et al., 2019). Therefore, to explore the effect of *Aa* exRNAs on NF-κB activation, we performed western blotting using anti-phospho-p65 antibody to detect phosphorylated p65, which is the active subunit of NF-κB. We found that removal of RNA cargo of OMVs by treatment with RNase reduced phosphorylation activity, whereas total p65 expression showed no apparent differences (Figure 5C). The overall findings indicated that *Aa* exRNAs promote the production of IL-6 in microglial BV2 cells through NF-κB activation.

## Discussion

Among the numerous bacteria involved in periodontitis, *Aa* is a small gram-negative bacterium previously well-known for causing localized aggressive periodontitis (Henderson et al., 2010). *Aa* is also implicated in various systemic diseases such as non-alcoholic fatty liver disease (Komazaki et al., 2017), diabetes (Demmer et al., 2015), and rheumatoid arthritis (Konig et al., 2016). Furthermore, recent evidence suggests that *Aa* and other oral microorganisms are associated with neuroinflammatory diseases such as AD (Laugisch et al., 2018; Dominy et al., 2019; Díaz-Zúñiga et al., 2019; Han et al., 2019; Wu et al., 2019). However, whether bacteria themselves directly or their derivatives are involved in diseases remains debatable (Lee, 2020).

Microglial cells are activated by various stimuli such as LPS, IFN-γ, and β-amyloid and also release proinflammatory cytokines such as IL-6, IL-1β, and TNF-α, which have been investigated with regard to AD (Wang et al., 2015). However, there is limited information regarding the effect of pathogenic exRNAs in microglial cells on proinflammatory cytokine secretion.

The meninges consist of three layers, the dura mater, the arachnoid mater, and the pia mater. The arachnoid mater is a membrane barrier that separates the dura mater from the remaining portion of the brain and has well-regulated junctions, similar to the BBB (Rua and McGavern, 2018). Studies conducted using CX3CR1-GFP transgenic mice reported a high density of meningeal macrophages in the dura mater and pia mater (Chinnery et al., 2010). Along with other CNS macrophages (perivascular and choroid plexus), meningeal macrophages are non-parenchymal immune modulators at brain boundaries (Goldmann et al., 2016; Rua and McGavern, 2018). Therefore, their immune response against infection could have pivotal consequences for CNS homeostasis and diseases. However, these macrophages have received little research attention with respect to oral pathogens and commensal bacteria in the context of brain pathogenesis due to the tight defenses of the CNS. Recently, considerable effort has been devoted to understanding tiny particles originated from bacteria (i.e., bacterial EVs) and their roles as messengers in human diseases given their ability to travel freely from cell to cell in the body (Choi et al., 2017b; Lee, 2019).

In the present study, we demonstrated for the first time, using intravital imaging analysis, the transport of OMVs from peripheral introduction to brain microglial cells through the meninges. The delivery of bacterial EVs into the brain after crossing the BBB suggests that infection at any place in the body can cause a fatal immune response in the brain. Since the number of human commensal bacteria has been estimated to be the same as the total number of human cells (Sender et al., 2016), bacterial EVs would be continually produced and the effect of chronic brain stimulation by bacterial EVs from commensal bacteria may be attributed bacterial OMVs crossing the BBB.

OMVs are taken up by host cells through various methods, and it is known that *Aa* OMVs are internalized into cells by membrane fusion or clathrin-mediated endocytosis (Rompikuntal et al., 2012; Thay et al., 2014). Our results clearly demonstrated that *Aa* OMVs and RNA cargo (i.e., exRNA) can enter into microglial cells (Figure 4).

It is known that LPS isolated from *Aa* has the ability to stimulate the production of TNF-α, IL-1β, and IL-6 in human whole blood (Schytte Blix et al., 1999) and microglial cells (Díaz-Zúñiga et al., 2019), however, little is known about the effect of other pathogenic factors of *Aa* on microglial cells. Because intact LPS of OMVs might have less effect than purified or released forms of OMVs, internalized OMVs and their cargo may activate various immune signals as cytosolic LPS activates caspase-11 and exRNAs enhance the TLR8 pathway (Vanaja et al., 2016; Han et al., 2019).

Interestingly, murine BV2 microglial cells reacted in a manner unlike human U937 macrophage cells upon treatment with *Aa* OMVs and exRNAs. Because we used a relatively low number (4.5 × 10^8^ particles) of OMVs compared to that in a previous study (4.5 × 10^10^ particles) to infect macrophage cells while avoiding BV2 cell damage, the lower dose of OMVs may be similar to the actual immune response that occurs *in vivo*. Among the various proinflammatory cytokines, IL-6 is the main player as it elevates the activity of proinflammatory signaling pathways and is associated with a large number of immune diseases (Luo and Zheng, 2016). Therefore, the increased IL-6 production in BV2 microglial cells by *Aa* exRNAs might be a novel causative agent of neuroinflammatory diseases.

Furthermore, our previous data showing that increased production of TNF-α by *Aa* OMVs in the mouse brain (Han et al., 2019) might be modulated by other cell types such as astrocytes suggests that activated microglial cells induce the secretion of TNF-α by astrocytes (Liddelow et al., 2017). As TNF-α stimulates the induction of IL-6 production as observed in brain pericytes and astrocytes and pericyte-originated IL-6 activated BV2 microglial cells (Matsumoto et al., 2018), various types of brain cells interact with each other via cytokines they produce in response to exogenous bacterial EVs and their cargo.

Taken together, our findings reveal a previously unrecognized mechanism of RNA transport in bacterial EV-based induction of brain immune response to peripheral bacterial infections. Further studies are necessary to investigate the range of bacterial EVs and exRNAs that are constantly produced and affect entire organs. It is also necessary to further explore strategies for preventing the chronic stress caused by bacterial EVs and exRNAs in relevant immune diseases.

## Data Availability Statement

The original contributions presented in the study are included in the article/Supplementary Material, further inquiries can be directed to the corresponding author.

## Ethics Statement

The animal study was reviewed and approved by the Institutional Animal Care and Use Committee (IACUC) of the Korean Advanced Institute of Science and Technology (KAIST, Daejeon, South Korea).

## Author Contributions

JH, S-YC, and JL contributed to the data collection and analysis. S-HH contributed to the data analysis and critically revised the manuscript. H-JL drafted the manuscript and contributed to the data collection and analysis as well as conception and design. All authors gave their final approval and agreed to be held accountable for all aspects of the work.

## Conflict of Interest

The authors declare that the research was conducted in the absence of any commercial or financial relationships that could be construed as a potential conflict of interest.
