# Supplementary material for: Delivery of Periodontopathogenic Extracellular Vesicles to Brain Monocytes and Microglial IL-6 Promotion by RNA Cargo
Source: Front Mol Biosci. 2020 Nov 24;7:596366. doi: 10.3389/fmolb.2020.596366 (PMC7732644; doi:10.3389/fmolb.2020.596366)
Supplement: Supplementary file 1 [file Data_Sheet_1.docx]

Supplementary Material

# Supplementary Data

**Table S1.** Sequences of primers used in this study.

| Gene | Primers (5’→3’) |
| --- | --- |
| β-actin | Forward: TGTCCACCTTCCAGCAGATGT |
|  | Reverse: AGCTCAGTAACAGTCCGCCTAG |
| TNF-α | Forward: GGCGTGGAGCTGAGAGATAAC |
|  | Reverse: GGTGTGGGTGAGGAGCACAT |
| IL-1β | Forward: TGGAGAAGCTGTGGCAGCTAC |
|  | Reverse: GAACGTCACACACCAGCAGG |
| IL-6 | Forward: TCAATGAGGAGACTTGCCTG |
|  | Reverse: GATGAGTTGTCATGTCCTGC |

**Table S2.** Cytokines array assay.

|  | **IL-1β (pg/ml)** | **IL-6 (pg/ml)^§^** | **IFN-γ (pg/ml)** | **TNF-α (pg/ml) ^§^** |
| --- | --- | --- | --- | --- |
| **Mock** | **< 0.55** | **9.25±0.59** | **< 0.59** | **28.73±3.29** |
| **OMV^a^** | **< 0.55** | **18.15±1.74** | **< 0.59** | **25.50±0.79** |
| **OMV^b^** | **< 0.55** | **21.75±1.63** | **< 0.59** | **28.49±0.64** |
| **OMV^a^lysate+DNase+RNase** | **< 0.55** | **12.81±1.29** | **< 0.59** | **24.58±0.44** |
| **OMV^b^lysate+DNase+RNase** | **< 0.55** | **14.78±2.64** | **< 0.59** | **25.19±1.06** |
| **OMV lysate^a^** | **< 0.55** | **17.66±0.70** | **< 0.59** | **26.95±0.63** |
| **OMV lysate^b^** | **< 0.55** | **21.11±0.77** | **< 0.59** | **26.75±0.76** |
| **OMV lysate+DNase^a^** | **< 0.55** | **18.42±1.14** | **< 0.59** | **27.25±0.66** |
| **OMV lysate+DNase^b^** | **< 0.55** | **23.75±3.33** | **< 0.59** | **28.77±2.12** |
| **OMV lysate+RNase^a^** | **< 0.55** | **12.22±0.46** | **< 0.59** | **22.61±1.94** |
| **OMV lysate+RNase^b^** | **< 0.55** | **11.8±0.74** | **< 0.59** | **24.03±0.68** |

*Aa* OMV and OMV lysates were treated on BV2 cells (5 × 10^5^ cells/2 ml in a 6-well plate) for 16 h (the levels of LPS were approximately 50 ng/ml in both OMV and OMV lysates). The OMV particles were around ^a^2.3 × 10^8^ or ^b^4.5 ×10^8^ particles.**^§^**The data are presented as the mean ± SD from three independent experiments. Cytokine levels of BV2 cells after treatment of intact OMVs or OMV lysates with nucleases (see main text).


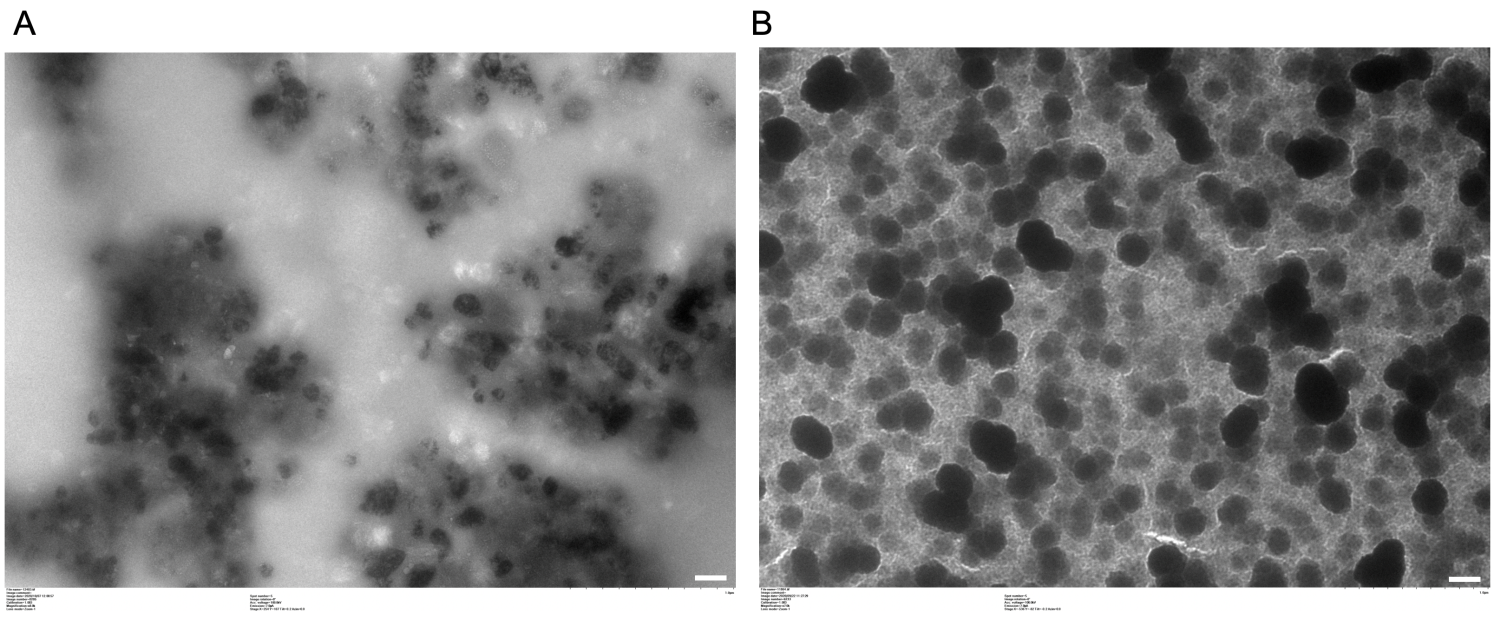


**Figure S1. Transmission electron micrograph (TEM) images of *Aa* OMVs.** Pre-purified *Aa* culture media (A) and purified OMVs (B) were compared. Both pre-purified culture media and purified OMV samples were diluted 10 times with PBS and applied to 200-mesh Formvar/Carbon grids (Ted Pella, Redding, CA, USA) without staining. Samples were dried overnight on the grids and viewed with an electron microscope (HT7700; Hitachi, Tokyo, Japan) operated at 100 kV. All panels are at original magnification, × 8,000. Scale bar, 200 nm.


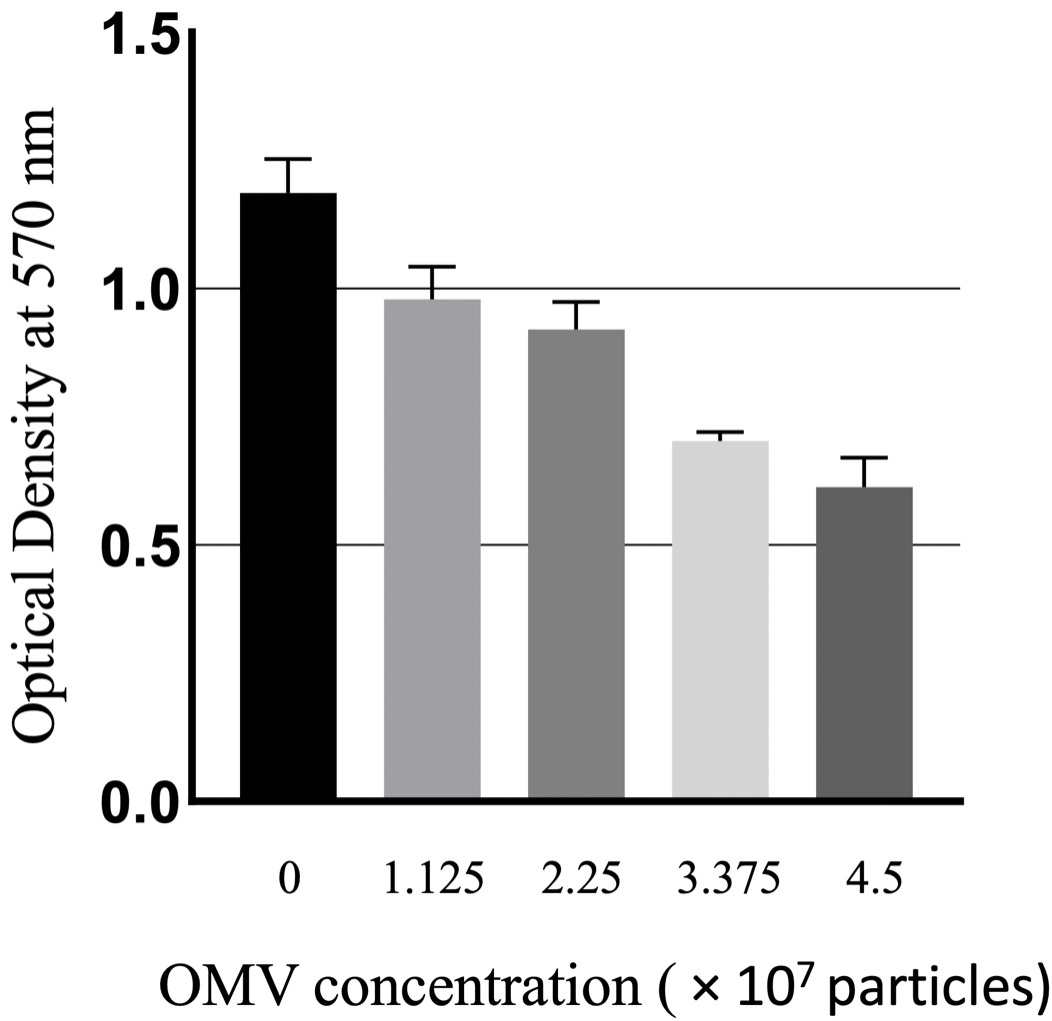


**Figure S2. Cell viability by *Aa* OMV.**To investigate the effect of *Aa* OMV on cell viability, BV2 cells were seeded onto 96-well plates (at a density of 5,000 cells/well). On the following day, cells were treated with different concentrations of *Aa* OMVs (1.125 - 4.5 × 10^7^ particles) in fresh medium and incubated for another 24 h. Cell viability was then assessed using the MTT (3-[4, 5-dimethyl-2-thiazolyl]-2,5-diphenyl-2H-tetra- zolium bromide) assay, and the absorbance was read at 570 nm using an ELISA microplate reader (Molecular Devices, Downingtown, PA, USA). These results are from three independent experiments; each bar represents standard deviation.

**
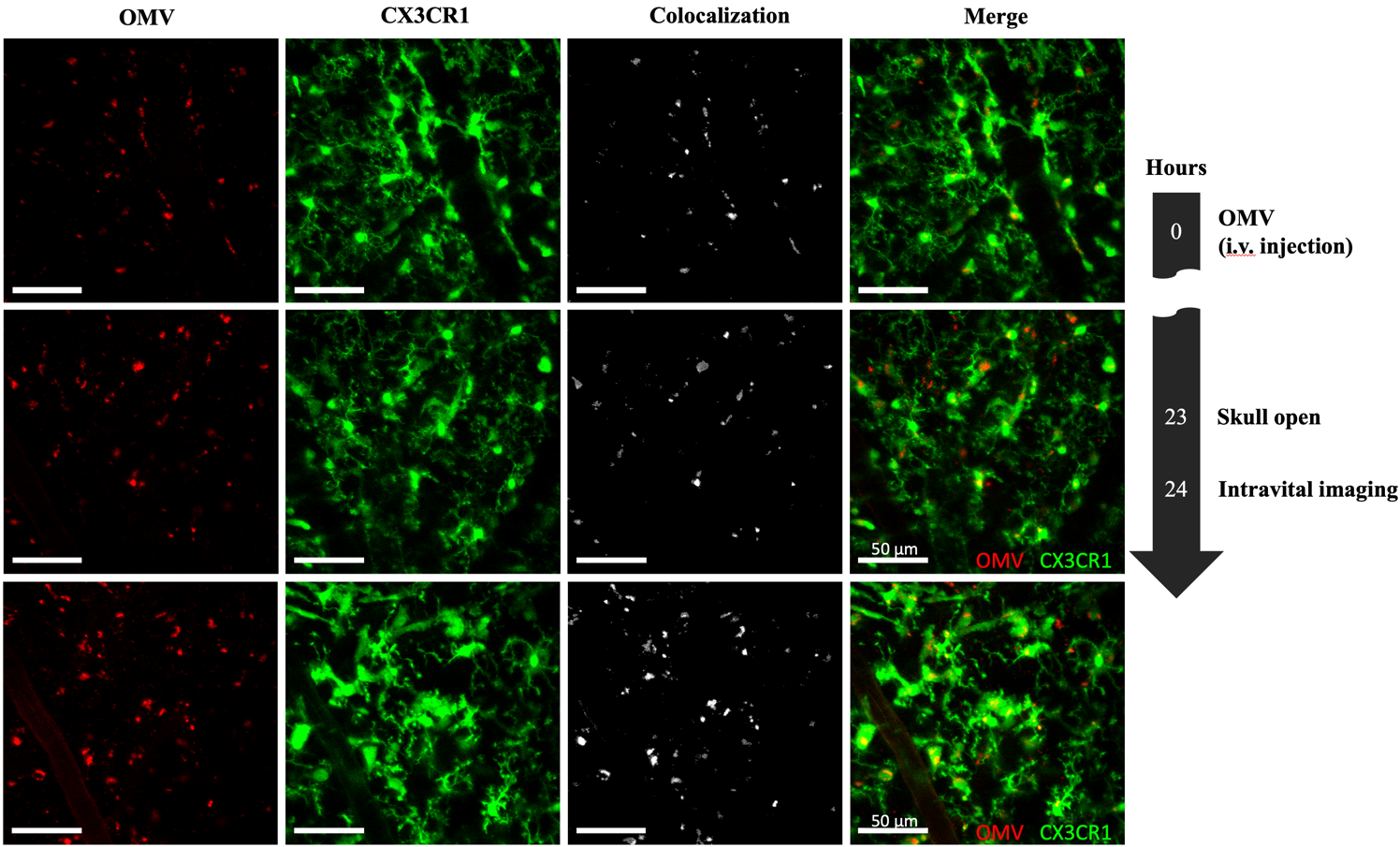
**

**Figure S3. Delivery of *Aa* OMVs into microglial cells (additional figures to Fig. 1C from different animals).** Intravital image was captured 24 h after *Aa* OMV i.v. injection. OMVs were colocalized with GFP-positive microglial cells. Scale bar: 50 μm.
